# Exploring constructions of female surgeons’ intersecting identities and their impacts: a qualitative interview study with clinicians and patients in Ireland and Scotland

**DOI:** 10.3389/fmed.2024.1379579

**Published:** 2024-07-22

**Authors:** Gozie Offiah, Stuart Cable, Susie Schofield, Charlotte E. Rees

**Affiliations:** ^1^Royal College of Surgeons in Ireland (RCSI), University of Medicine and Health Sciences, Dublin, Ireland; ^2^Centre for Medical Education, School of Medicine, University of Dundee, Dundee, United Kingdom; ^3^School of Health Sciences, College of Health, Medicine and Wellbeing, The University of Newcastle, Callaghan, NSW, Australia; ^4^Faculty of Medicine, Nursing and Health Sciences, Monash Centre for Scholarship in Health Education, Monash University, Clayton, VIC, Australia

**Keywords:** gender, identities, surgical training, surgical education, intersectionality

## Abstract

**Introduction:**

While many studies have explored surgeons’ personal and professional identities separately, our study is the first to examine intersecting female surgical identities. We explore intersecting surgical identities constructed by self and others (colleagues and patients) within two healthcare systems and their perceived impacts answering the research question: How do female surgeons’ constructed identities intersect, and what influences do those intersections have on their surgical lives?

**Methods:**

We employed qualitative methodology drawing on semi-structured biographical narrative interviews underpinned by social constructionism. We employed intersectionality theory as an analytical lens. We adopted maximum variation sampling to identify diverse participants, including 38 surgeons (29 female; 9 male), 9 non-surgical colleagues (8 female, 1 male), and 13 patients of female surgeons (7 female, 6 male). Our 29 female surgeons also included six who had transitioned out of surgery. We analyzed the dataset using a five-step Framework Analysis approach. We captured talk *about* identities, as well as identity talk (constructions of identity through participants’ talk).

**Findings:**

Multiple intersecting personal (not just gender) and professional identities were constructed and reported to have multiple impacts on female surgeons’ lives (including their education, training, and success). We present intersecting identities and their impacts relating to gender through four primary intersections: (a) gender + ethnic identities; (b) gender + parenthood identities; (c) gender + age identities; and (d) gender + professional identities (namely carer, competent, mentor/mentee, role model and leader). Our findings particularly highlight the challenges experienced by female surgeons of color, who are mothers, who are younger and/or who are leaders, illustrating diversity in women’s experiences beyond that of gender alone. Finally, we found unexpected insights into male surgeons and fatherhood.

**Discussion:**

Intersectionality theory offered a novel analytical lens to extend existing knowledge on female surgical identities. Further research is warranted exploring intersecting identities of female surgeons of color, younger female surgeons, and male surgeons who are fathers, as well as identities unexplored in this study (e.g., diverse sexualities). We need to raise awareness of intersecting identities and their impacts in surgery, as well as providing training, allyship, and policy revision that is sensitive to intersectionality.

## Introduction

A significant body of evidence demonstrates that women face specific workplace challenges, adversely impacting the female medical workforce (1–3). Despite the higher number of females in medicine nowadays, there are still few women in professionally influential areas such as surgery leadership and clinical academia (4). There has been a push to increase the number of women in the medical workforce, thus creating pressure for more flexible career paths to manage work interruptions (5). Indeed, changes in lifestyle, career transitions, and working environments, alongside this feminization of the workforce, have increased the demand not just for flexible training but also for flexible working within the surgery field (6). With such workforce changes, flexibility will become increasingly important for women and men, as per the consensus statement from The Association of Surgeons in Training (7). However, very little has been published to date within the context of the medical workforce exploring female gender (including gender bias and stereotyping) with other intersecting personal and professional identities.

### Intersectionality theory

Intersectionality theory was introduced by Crenshaw, an American civil rights advocate, in 1989 (8). She used the term to describe the intersection of individuals’ different identities (9). Personal identities, such as gender, ethnicity, social class, nationality, religion and age, can intersect with professional identities (10). The theoretical framework of intersectionality postulates that multiple social categories, including ethnicity, gender, sexual orientation and socioeconomic status, intersect at the micro-level of individual experience to reflect multiple interlocking systems of privilege and oppression at the macro, social-structural level to include things like racism and sexism (11). Intersectionality theory suggests that no one identity (for example, gender) is prioritized over another (for example, ethnicity). It emphasizes that gender may or may not be a priority for women when constructing their multiple identities. Individuals may understand identity by combining a person’s traits and interactions that shape their experiences of gender inequalities in power hierarchies (12, 13). The intersectionality lens provides a framework to conceptualize the multiple intersecting personal and professional identities interplaying for female surgeons’ lived experiences of gendered surgical environments. Women experience disadvantage and discrimination based on their sex and gender, which is inextricably linked to other identities, factors and experiences, such as race (14). While intersectionality theory has been discussed in the context of medical education to explore intersecting identities (4, 15–21), to our knowledge, none of the surgical literature has so far employed intersectionality theory to explore female surgeons’ intersecting identities. We use this theoretical lens to examine how gender intersects with other personal and professional identities to shape female surgeons’ experiences (including their reported experiences of gender discrimination and inequality) impacting their surgical careers.

### Gender and personal identities in surgical careers

Gender stereotypes based on traditional social roles of men as breadwinners and women as household caretakers shape general expectations for the types of jobs men and women should occupy (22). Additional personal identities include ethnicity, parenthood, and age. In terms of ethnicity, compared to white women, women of color^1^ are more likely to be segregated into less desirable, lower-paying jobs, such as domestic helpers, agricultural employees and factory workers (22). Moreover, as social power has been denied to women of color, they must learn how to negotiate power when in leadership positions (14). The literature so far has mostly focused on gender and ethnicity, with studies exploring the challenges for medical students, surgical trainees or other university students of color (23–25). For example, a recent qualitative study of US physician-trainees considered underrepresentation in medicine, suggesting that students of color must actively work to dismantle harmful stereotypes to align their racial identities with their professional (physician) identities (24). Residents of color in Canadian surgical programs have, for example, reported their competence being questioned more often than their white gender-matched peers. They also reported feeling that their reports of discrimination would not lead to appropriate actions being taken (24). While this study represents an important recent addition to the literature, the study was based on survey findings with a 40% response rate from general surgery residents only. Therefore, the survey responses may have been collated from participants with negative experiences of diversity during surgical training only, thus skewing the data.

Regarding motherhood, other studies have explored its impacts on work-life balance, reproductive outcomes and career progression (4, 26–28). Historically, the structure of surgical programs can discourage women interested in both surgery and motherhood from pursuing a surgical career due to the perceived tensions between motherhood and surgical careers. Hill et al. explored the identity formation of female surgeons (27). They interviewed 15 female surgeons and argued that being female identified them as “other” within surgery, and as such, they were required to demonstrate masculine traits to gain legitimacy in the surgical world (27). It is noted that this cross-sectional study involved small numbers of interviewees across each stage of training—five medical students, three consultants^2^ and one retired consultant. At the same time, the authors in that study described it as “world-making,” which they referred to as a discursively powerful phenomenon, enabling women to re-narrate their feminine and surgical identities as mutually compatible, building upon each other in a complementary and strengthening way (27). While Hill et al. and other studies outside of medicine have shown that mothers can be penalized on several measures, including perceived competence and financial rewards (29, 30), none has explored the perceptions of female surgeons of all grades, nor colleagues and patients of female surgeons about surgical + motherhood identities. Although our study set out to explore women and surgery, we also present in this paper new (and unexpected) findings relating to surgery and fatherhood to serve as a counterpoint to female surgeons’ experiences.

Finally, concerning age, several studies have explored surgeons’ age and their surgical proficiency or the outcomes of surgery; for example, a recent JAMA paper assessing the performance of aging surgeons stated that surgeons’ competence should be based on functional age and abilities rather than chronological age (31). A few studies have explored gender and age intersections in surgeons, focusing on the impact of patient mortality. Tsugawa et al. showed that patients treated by older surgeons had lower mortality rate than patients treated by younger surgeons, but patient mortality did not differ between male and female surgeons (32). A study of patients’ preferences for their surgeon based on the gender and age of the surgeon showed that female patients had a preference for female urologists while male patients expressed no preference for the gender of their urology surgeons, and this gender preference did not correlate with age (33). Over the last 10 years, only one study has explored intersecting gender and age identities, exploring women surgeons’ concerns about being too old after residency to have a child (34). Their study focused on medical students’ perceptions of barriers in surgery, surveying 720 medical students using a questionnaire. The authors found that more female than male students reported concerns about time to date or marry and being too old after residency to have a child; these concerns deterring more women than men from surgery. Despite these recent studies, there is still a lack of literature on gender and age intersections in surgery; hence the importance of exploring such intersecting identities within this current paper.

### Gender and professional identities in surgical careers

Professional identity is part of a broader social identity, which can also depend on a person’s cultural background, such as ethnicity and religion (35). Every doctor will have professional identities shaped by and shaping their social identities. For example, cultural overlays, like relationships between doctors and nurses, can be hierarchical in some cultures and egalitarian in others (36). Professional identity has been defined variously in the literature, including as the attitudes, values, knowledge, beliefs and skills shared with others within a professional group, with the development of professional identity being a continuous process (37, 38). It has also been associated with the commitment to perform competently and legitimately in the context of the profession, and proposed to be a requirement for taking up professional responsibility, and this self-perception is separate from any external indicator of professional preparedness (39, 40).

We examined the literature for studies around professional identity in surgical careers. Several studies have focused on the development of surgical identities as journeys, but others have explored surgical identities around communities of practice and interprofessionalism (41, 42). Other studies have redefined and challenged the traditional stereotype of the surgeon as egotistical, paternalistic and inflexible, and instead focused on surgeons as role models and coaches (43–45). Others have focused on the formation and modeling of surgeons’ identities, which starts prior to training and lasts throughout their lives, influenced by professional experiences, interpersonal relationships, individual factors and external influences (46). However, none of these studies has explored any intersecting professional and personal identities of surgeons.

Mobilio et al. explored the dual identities of surgeons and learners in the operating room (47). They discussed autonomy as a critical component of a surgeon’s professional identity. The need for female surgeons to internalize a leadership identity through education, role modeling and mentoring has also gained recognition in recent years, highlighting the need for more female role models and mentors in surgery; challenging still due to low numbers in surgery (especially in some surgical specialties). Hill and Vaughan argued that the inability to see or identify with other women in surgery deters female students from pursuing careers in surgery (48). Studies have identified a particular challenge in transitioning to consultant surgeon leadership positions (26). This study exploring higher-stage trainees’ transitions into consultant positions included a female surgeon’s journey as a longitudinal case study and how the female surgeon found the transition to consultant and her leadership identity a struggle. The female surgeon discussed how she: “felt a bit of a fraud being called the consultant,” and the impact of this on her relationship with work colleagues. This perceived sense of self-doubt, combined with fear of being exposed as a fraud (so-called imposter syndrome), is very prevalent in general surgery training culture and is well documented in the literature (49–56).

Finally, while much has been published about female surgeons in male-dominated environments, very few studies have explicitly explored male surgeons’ intersecting personal and professional identities. Most studies on male surgeons focus on postoperative outcomes and operating times, showing that patients operated on by male surgeons had more surgical complications and total complications than female surgeons (57, 58). However, as will be revealed in our findings below, our study provided a unique and unexpected opportunity to illustrate perceptions of male and female surgeons, colleagues, and patients of female surgeons on the intersecting identities of male surgeons. We include these findings in this female-focused paper as a counterpoint to the female experience; to better understand it comparatively. As mentioned above, while studies have explored identities previously, more research using intersectionality theory is needed to examine the diversity of surgeons’ intersecting personal and professional identities. We think that this paper provides these new insights, and also shows the need for more work exploring intersectionality in surgery.

### Study aims and research questions

Our study set out to address a gap in the literature about constructions of female surgeons’ intersecting personal and professional identities, providing novel insights into how female surgeons navigate the surgical environment and their surgical careers. As mentioned above, we not only wanted to hear from female surgeons themselves, but we also wanted to hear from other stakeholders (i.e., male and female colleagues and patients) to elicit a novel and comprehensive picture of female surgeons from multiple perspectives. However, we also yielded novel (and unexpected) findings regarding male surgeons (especially around fatherhood), so these are also included in the paper for reasons of completeness and comparativeness. We conducted this gender-focused study in Ireland and Scotland. Using *Intersectionality theory* as an analytical lens, our paper therefore aims to answer the following research question (8):

- How do surgeons’ constructed identities intersect, and what influences does this intersectionality have on their surgical lives?

## Materials and methods

### Study design

We employed a qualitative methodology drawing on face-to-face and online semi-structured biographical narrative interviews underpinned by social constructionism (59). As discussed above, we employed intersectionality theory as an analytical lens (8). Biographical narrative interviewing provides a relatively coherent “whole story” approach, with many personal incident narratives within that whole story/long narration (60). Biographical narrative interpretive interviewing was employed for our study as it emphasizes the socially constructed nature of reality and helps to provide a rich and complex understanding of female surgeons’ experiences from multiple perspectives (61).

### Context

Our study was conducted with participants situated within two healthcare systems—Ireland and Scotland (which have similar postgraduate training programs for surgeons). Both run a 6–8-year surgical training program with 2-year core surgical training and then a specialist training program, which varies from 4 to 6 years depending on the surgical subspecialty. The final 3 years are individually tailored to sub-specialty training according to the Intercollegiate Surgical Curriculum Programme (ISCP) (62). Our study focused on female consultant surgeons and higher-stage surgical trainees. The surgical trainees were beyond the halfway point of their higher speciality training programs to ensure female surgeons had spent at least 5 years in surgery, and thus data collected was fully reflective of the lived experiences of female surgeons. At the time of the study, female surgeons in Ireland and UK respectively, were roughly 10 and 16.3% of all surgeons (63, 64).

### Sampling and participants

After receiving ethical approval from six sites in Ireland and one overarching approval in Scotland (see ethics section for details), we purposively sampled participants through emails, posters, snowballing, and a clinical reference group (see acknowledgements section) across six hospital groups in Ireland and seven health boards in Scotland. Our purposive sampling served to include primarily female surgeons (with diverse other identities such as age, ethnicity, nationality, and motherhood), as well as their male and female colleagues from any profession and/or speciality (including surgery, medicine, and nursing), and male or female patients whom they had recently treated within the last 2 years. This maximum variation sampling identified diverse constructions of surgeon identities from 38 surgeons (i.e., 29 female and 9 male), 9 non-surgical colleagues (3 anesthetists, 4 nurses, and 2 physician associates) and 13 patients of female surgeons. Female surgeons were identified across 9 surgical specialties with plastic surgery and orthopedics being the top two specialties represented. Table 1 illustrates the demographic characteristics of our participants in both countries.

**Table 1:** Participant demographics.

Female and male surgeons (*n* = 38)	
Country	
Ireland	18
Scotland	20
Gender	
Female Male	29 9
Ethnicity	
White Middle Eastern Asian	31 3 4
Age range	
18–29 30–39 40–49 50–59 60–69	3 16 13 5 1
Specialties	
Breast surgery Colorectal surgery Otolaryngology General surgery General trauma Neurosurgery Orthopedics Pediatric surgery Plastic surgery Transplant surgery Upper gastrointestinal surgery Urology Vascular surgery	5 3 1 6 1 1 5 2 7 2 2 1 2
Non-surgical colleagues (*n* = 9)	
Country	
Ireland Scotland	6 3
Gender	
Female Male	8 1
Ethnicity	
White	9
Age range	
18–29 30–39 40–49 50–59 60–69	2 2 1 3 1
Specialties/Professions	
Anesthesia Physician Associates Nurses	3 2 4
Patients (*n* = 13)	
Country	
Ireland Scotland	10 3
Gender	
Female Male	7 6
Ethnicity	
White	13
Age range	
30–39 40–49 50–59 60–69 70+	4 2 2 4 1

### Data collection

The same researcher (GO) conducted 60 individual, semi-structured interviews between November 2016 and April 2019, employing biographical narrative interviewing (61). These provided a rich and complex canvas, illustrating the constructions of surgeons’ intersecting identities. Before the interview, all participants completed a personal details questionnaire to elicit their demographics. The interviews began with an open-ended question, where surgeons were asked to recount their lives as a surgeon and non-surgical colleagues and patients were asked to recount their experiences with an (anonymous) female surgeon of their choice. Supplementary material provides the interview questions for female surgeons. Other interview schedules can be requested from the corresponding author or found elsewhere (65). All interviews were audio-recorded with permission, and the transcribed audio files assigned unique identifiers for each participant to maintain their anonymity. The 60 individual interviews provided sufficient *information power* (66), ranging from 18 to 76 min (average of 35 min), totaling 36 hours of rich, in-depth interview data.

### Data analysis

Our data analysis began in May 2017 and was completed by December 2020; so was partly sequential to data collection. The first author (GO) imported interview transcripts and related notes into NVivo version 12, where we (GO, SC, SS, CER) carried out an inductive process to identify and code key themes. We analyzed the dataset using a five-step Framework Analysis approach, which is a type of thematic analysis (67). The five steps included:

(1) *Familiarization*: working alone, we familiarized ourselves with a sub-set of interviews, reading the transcripts and listening to the interview recordings, and thinking about preliminary themes based on the data.
(2) *Framework development*: we got together to discuss and agree on our preliminary themes to develop our coding framework. We produced a final 18-page coding framework with five main themes (available on request from the corresponding author) and part of a broader program of research, with further details elsewhere (65).
(3) *Indexing*: using the coding framework, the first author coded all data using NVivo, with further developments to the coding framework occurring throughout this indexing process.
(4) *Charting*: we interrogated the data coded to themes and sub-themes to make sense of it. Our previous paper (6) explored the theme of career transitions; but in this current paper, we focus on the (previously unpublished) “Identities” theme, which we explored by examining talk *about* identities and identity talk (i.e., constructions of identity through participants’ talk).
(5) *Mapping*: we compared our findings with intersectionality theory and relevant literature. Key themes are presented in this paper, with illustrative quotes reflecting the construction of female surgeons’ intersecting identities and their impacts.

### Qualitative rigor

We established qualitative rigor via a team reflexivity exercise, which acknowledged differences among our perspectives, backgrounds, positioning and experiences, enabling a more thorough and diverse understanding of the data, thereby contributing to our rigorous analytic process (68). Our team included three females and one male. Two of us have clinical backgrounds; the other two are non-clinical health professions education experts. We range in our level of experience with qualitative methodologies and gender research (one expert, two intermediates and one novice). We purposely set out to achieve internal coherence by ensuring that our philosophical approach (relativist ontology, constructionist epistemology) aligned with our chosen theoretical framework (intersectionality theory), and the design of our methodology (interpretivist qualitative methodology) and methods (e.g., biographical narrative interviews); so there were no areas of incongruence (69, 70).

## Findings and discussion

**Figure 1:**
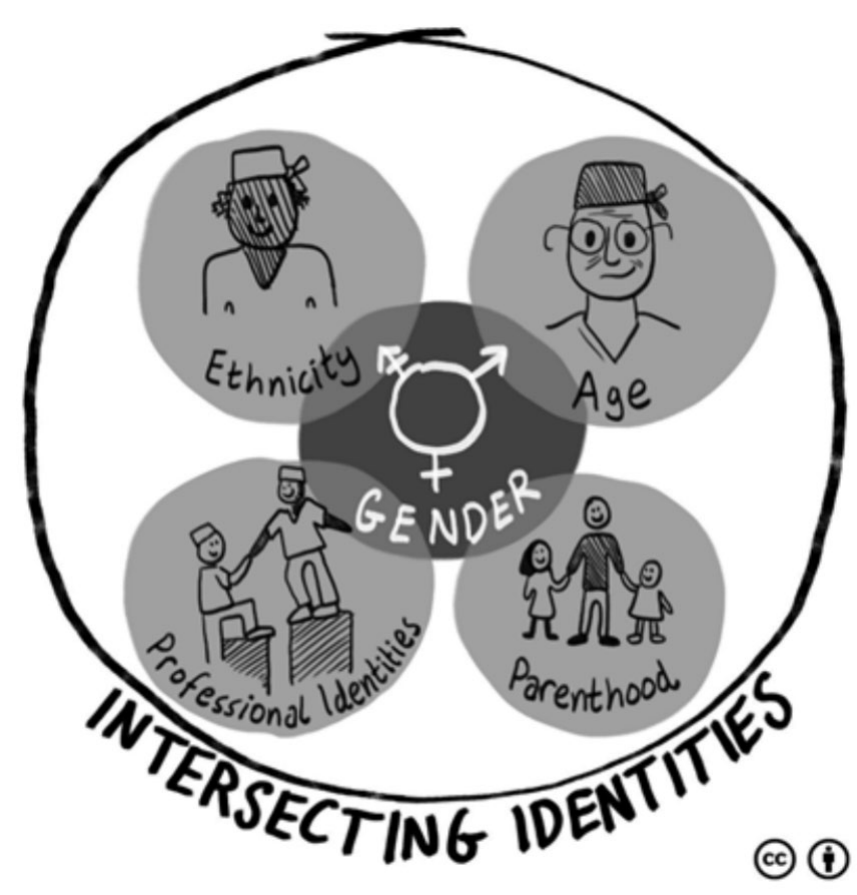
Illustration of the interplay of intersecting identities.

We explore here the narratives of all participants (not just female surgeons), illustrating what and how multiple constructed identities intersect within the surgical environment. The multiple identities (not just gender) played a crucial role in both female and male surgeons’ co-constructed identities and their perceived impacts on how they succeeded in their careers. In this paper, we illustrate the omnipresent influence of gender through its intersections with ethnicity, parenthood, age, and professional identities (see Figure 1). Not only do we illustrate these constructed intersecting personal and professional identities, but we also highlight how these intersections are seen to play crucial roles in surgeons’ lives (including their education, training, and success) from the perspectives of female surgeons and other clinicians and patients (of both genders). We combine our findings and discussion of findings in light of theory and literature in one blended section in this paper; as is acceptable for qualitative research (71, 72). We do this for three reasons: (1) to help facilitate integration between our presentation and interpretation of study findings; (2) to present a more parsimonious approach to presentation and discussion of study findings (thereby avoiding repetition of results across multiple sections); and (3) to reduce the reader’s cognitive load.

### Intersections between gender and ethnic identities

Female surgeons of color explicitly constructed their intersecting gender + ethnic identities, as well as its impacts on surgical careers. Female surgical trainees of color who were non-Irish (in Ireland) or non-British (in Scotland) seemed to have more challenging times in the workplace beyond being female; as indicated by the following white female surgical trainee:

*I think nationality, for me and for other people, does bear out in the NHS [National Health Service]… in terms of the trainees… some of us are white and British and some of us are not… the trainees that are non-white and non-British definitely get a harder time added onto the fact that they are also female… that’s quite difficult to witness… I do not think that being female is the only potential issue or source of bias in surgical training. (ID 22,* Scotland-based Female *Surgical Trainee, Aged 30–39).*

This female surgeon identified gender + ethnicity intersections, describing how women of color are treated differently in surgery. Her quotation illustrates that intersecting identities of female and non-white can lead to further inequalities in training experiences. Female surgeons of color also described how their intersecting identities could influence interactions with patients, as indicated by a resigned^3^ female surgeon of color:

*You know, there were two things that were putting her [the patient] off… One was my headscarf … and the other was… she thought I could not speak English … because of my dressing as opposed to my qualifications. (ID 38, Ireland-based Resigned Female Surgeon, Aged 30–39).*

This female surgeon narrates a patient judging her competence negatively based on her ethnic clothing and language. Female surgeons of color repeatedly described negative patient experiences around personal (gender + ethnicity) and professional identities (specifically competency). While this was common for all surgeons of color, participants reported this being more dominant for female surgeons of color (24). These findings align with intersectionality theory (8), emphasizing the unique ways in which women of color experienced their gender + ethnicity intersections, compared with women identifying as white. Our findings highlight that gender and racial/ethnicity biases impact female surgeons negatively throughout their training; affecting their career progression (23). Although white women and women of color are both subjected to societal gender norms, for women of color, these gender norms appear amplified through the lens of ethnic inequalities; which is consistent with existing literature (76). While such gender + ethnicity intersections and their impacts on the self and general mental health reflect other research (25), our study is the first of its kind to explore this intersection within Scottish and Irish surgical communities.

### Intersections between gender and motherhood identities

Some participants narrated challenges about being a female surgeon and parent, with motherhood perceived as a barrier to career progression. Participants narrated how becoming a mother and having maternity leave derailed the speed of their surgical career progression, as illustrated by the following resigned female surgeon and mother:

*Then I went and had a baby, so I was off for 6 months, and I just kept getting further and further behind, and then it eventually ended up with people who had been my SHOs [senior house officers] when I was a registrar, they were getting their consultant jobs, and that really hurt … (ID 31, Scotland-based Resigned Female Surgeon, Aged 40–49).*

This female surgeon explains how painfully she experienced the negative impacts of motherhood on her career with her juniors becoming consultants before her—using emotional talk (“*that really hurt*”). While several participants in our study narrated how they tried to keep their female surgeon + mother identities separate, others described their female surgeon + mother identity intersections, highlighting the sacrifices that female surgeons (and their partners) make for women to be both surgeon *and* mother:

*I have to say, I had a very supportive husband through my surgical career but being on-call … It was hard… I remember even my husband used to bring the kids [to the hospital] for me to breastfeed them at work … (ID 53, Ireland-based Female Consultant Surgeon, Aged 40–49).*

This female surgeon narrates the sacrifices she and her husband made to prioritize her career. Her breastfeeding was only made possible by her husband bringing her children into work so she could feed them and work simultaneously. In general, working mothers face multiple disadvantages (e.g., salary penalties, selection/recruitment barriers, perceived as less competent and committed), beyond those based on gender alone—a phenomenon called the motherhood penalty (77). In addition to the personal challenges expressed by female surgeons who were mothers, others talked about the negative impacts of motherhood on relationships with workplace colleagues. The following female consultant describes a lack of acceptance of motherhood and part-time surgical work among some senior male surgeons, with consequent negative impacts:

*… another friend of mine who has done Vascular Surgery who has a kid and came back part-time, her boss thought she was going to be in, but it was one of the days where she wasn’t working, and he phoned her and said, ‘Well where are you?’. She said, ‘Well, actually, I’m not at work today… It’s one of my [non-work] days’… he… said to her, ‘Well, I hope you are a better mother than you are a surgeon’. (ID 43, Scotland-based Female Consultant Surgeon, Aged 30–39).*

This demonstrates the negative perceptions held by some colleagues about part-time working mothers in surgery. Here, the consultant alludes to this part-time female surgeon’s lack of competence and commitment as a surgeon, but also her (in)competence as a mother. These negative stereotypes about working mothers are challenging for female surgeons as they navigate the demands of their working parenthood (77). While our findings highlight participants’ talk of female surgeon + mother intersecting identities, others talk about the incompatibility between motherhood and surgery (27). Although Hill et al. allude to the separation between motherhood and surgeon identities, our novel findings demonstrate female surgeons’ intersecting mother + surgeon identities and the associated sacrifices (27).

### Intersections between gender and fatherhood identities

Despite setting out to explore female surgeons’ experiences, we also found constructions of male surgeons’ fatherhood identities, as perceived by female surgeons, colleagues and patients. We include these novel and unexpected findings here as a counterpoint to female surgeons’ experiences. Interestingly, female participants’ data implied that being a father did not negatively affect male surgeons’ career advancement (unlike for female surgeons + mothers). The following female surgical trainee believes that male surgeons can more easily delay having children until training completion:

*Males can wait longer to have kids than females, so females at the same age range might be trying to have kids if they are in surgery, whereas males can wait ‘til they are 38, 39, 40 to have their first kid. (ID 66, Ireland-based Female Surgical Trainee, Aged 30–39).*

She argues that fatherhood is not a barrier to surgical career progression because male surgeons have more flexible fertile years, implying that male surgeons might not have the same work and/or parenting penalties found in female surgeons (28). However, contrastingly, both younger male surgical trainees and older consultants in our study reported their dismay regarding the negative impacts of surgical cultures on their family lives:

I was faced with working in [names city #1] while my wife was in [names a different city #2], 36 km away. My son was three months old; my daughter was three years old, and I thought, either we do it, or I become a GP. And so, I did it [worked in a different city], I do not know how we did it, but we did it… I had four and a half years [being apart], she [wife] got an inter-deanery transfer and came to [names city #1]. So, then we were together again… I used to get in a car on a Sunday evening to drive down… driving down the motorway, I’d have tears in my eyes because… every third weekend I was away for two weeks, and I did not see them for two weeks… *(ID 24, Scotland-based Male Consultant Surgeon, Aged 50–59).*

He expresses the emotional challenges of balancing his family life with work and his conflicting motivations (i.e., staying in surgery, and being a good father/husband). We see this male surgeon report being away from his family and children for 4.5 years to progress his surgical training. We might infer that his wife looked after the kids despite her medical job during his absences, highlighting how male doctors’ careers can be prioritized within medical couples (78). She appears to make sacrifices in her career through an inter-deanery transfer to be near her husband. Previous literature has also focused on the implications of work-life balance and its impact on the relationships between medical couples (79–81). Interestingly, other (younger) male surgeons expressed wanting more equality for male surgeons in terms of flexible working, so they could be more hands-on dads, with the perceptions that male surgeons were sometimes disadvantaged:

*I’ve got no problems with a year off maternity leave… it should be mandatory for all men as well. If I have a new-born child, I should be offered a year off as well. You know it should be total equality … you hear old chauvinists in the past going on about ‘Oh the women should not get time off to look after their kids’, that was the reason why women would not go into surgery. It’s absolutely ridiculous. But everyone should be equal … there was a lot of time that my wife would have appreciated me at home… (ID 40, Scotland-based Male Consultant Surgeon, Aged 40–49).*

This male surgeon expresses the need for an equal system supporting parental leave for all. He describes how his wife would have benefitted from him being at home, which would have enabled him to embrace his fatherhood identity. While Morgenroth et al. suggests that gender and fatherhood identities were privileges and that men are awarded fatherhood advantages, our findings suggest that male surgeons view surgical cultures as disadvantageous to accomplishing their fatherhood + surgeon intersections (like female surgeons) (30).

### Intersections between gender and age identities

Female participants narrated experiences of being subjected to “gendered talk” relating to their ages, as illustrated by the following female surgical trainee who narrates her multiple intersecting personal (age + gender) and professional identities (incompetent surgeon, surgical trainee):

*I think the older generation [of patients] tend to … I think their first perception is that you are not a surgeon. And when you do say you are a surgeon, it becomes a question of ‘oh but you’re very young’ or ‘are you sure you know what you’re doing? Is there going to be someone with you in surgery?’ It’s usually ‘are you being supervised?’ (ID 21, Scotland-based Female Surgical Trainee, Aged 30–39).*

Several female surgeons reported age biases from older patients, particularly indicating a lack of confidence in young surgeons’ competence (equating youth with a lack of knowledge/experience). Participants made distinctions between individuals’ age identities by using “older” or “younger” as proxies for “experienced” or “inexperienced.” The intersecting gender + age identities seem to play a crucial role in female surgeons’ surgical careers. For example, younger female surgeons narrated more powerlessness in their interactions with patients and colleagues, with some alluding to imposter syndrome, as has been indicated in previous research (49–55). Such imposter feelings were sometimes evident in female surgeons’ narratives, expressing a lack of support from their colleagues, as suggested by the following female surgical trainee:

*This was probably my first feeling of being a total imposter … I applied [for surgical training] because I was encouraged to do it and then was told by my consultant that it would actually be a really bad idea if I got on because I was quite young… that I was just a bit naive. (ID 65, Ireland-based Female Surgical Trainee, Aged 30–39).*

This 30-something female trainee described the previous lack of support from her (then) consultant, who tried to dissuade her from pursuing surgical training because she was “quite young” (and ipso facto “naive”). Interestingly, we found multiple examples in participants’ talk where female surgeons described their infantilization in the workplace:

*… in my first rotation as an SHO, I got pulled aside by my registrar, who said, ‘the consultant appreciates it when girls look like girls, think about how you dress and appear at work’. (ID 22, Scotland-based Female Surgical Trainee, Aged 30–39).*

This female trainee reports how she was infantilized by her surgical registrar—being called a “girl,” as well as being told to dress in a gender-concordant manner (“girls look like girls”) to please her male surgical consultant. This serves simultaneously to physically objectify her and reify her lower status in this mixed gender training relationship with a male surgical consultant. Sadly, dominant in our data were male surgeons often referring to their female colleagues as “girls,” while female surgeons rarely used this term to refer to female colleagues. In many instances, the term “girls” served to deprofessionalize female surgeons (82). Our findings align with other literature showing that women are less often introduced by their title, and are commonly mistaken for non-physicians (83, 84).

Interestingly, participants conceptualized intersecting gender + age identities differently for male surgeons; another noteworthy counterpoint to female surgeons’ experiences. One female surgical trainee described a young male consultant as “drunk on power,” with dominant constructions found in our data of competent surgeon + male + young identities:

*I think he’s a young enough consultant, probably a little bit drunk on power; you know, this kind of malarkey. And people telling him that he’s great. (ID 65, Ireland-based Female Surgical Trainee, Aged 30–39).*

Despite constructing this young male consultant derogatorily as an authoritarian figure, this female surgeon explains how his gender + youthful age is supported in the surgical system, serving to construct his professional identity as competent. So, while youth is perceived as disadvantageous for female surgeons (they are perceived as too young/inexperienced/incompetent), it is not perceived as negatively for young male surgeons (who are perceived as doing great for their age). Furthermore, older male surgeons are narrated as being more experienced/competent in our data, but we did not identify this issue in our dataset for older female surgeons. In the following quotation, we provide an example from a 50-something female patient, illustrating the intersecting personal (male + older) and professional identities (competent surgeon):

*I think he’s more experienced… he understood it a little bit more because he was more mature… Seniority brings competence; I think so, yeah. Yeah, well, it kind of made you feel comfortable… you know by looking at him he’s good at what he does because he’s got the confidence and age on his side, and you go ‘well he’s been here for a while, so he’s obviously very good’. (ID 75, Ireland-based Female Patient, Aged 50–59).*

Here, the patient describes how she felt the male surgeon understood her more because he was older (“mature,” “seniority,” “age on his side”), which she equates with confidence and competence (including just by “looking at him”). This male + older privilege stands in stark contrast to the findings discussed earlier (female + youthful disadvantage), where patients query young female surgeons’ competence.

### Intersections between female gender and professional identities

As above, the intersections between gender and professional identities were dominant across participants’ narratives. The following female consultant surgeon describes her first encounter with an 8-year-old male patient who was bemused that the doctor was female:

*I had a little one a few months ago, and he could not understand that I was a doctor, and he said to his mum… ‘but she’s a woman’ (laughter)… he must have been, oh, eight or something and his mum was like ‘Yes, women can be doctors too’, and he was like ‘No, no but she’s a woman’ (laughs) which was quite amusing. (ID 43, Scotland-based Female Surgeon Consultant, Aged 30–39).*

Professional + female gender identities clearly created tensions for this young male patient. Although this female consultant recounts this experience with laughter and states that she found it “amusing,” this example signifies strongly the (still) prevailing societal expectations about gender roles: that doctor equals male (3, 85, 86). The professional identities further explored in this section include intersections between female gender and specific professional identities such as: carer, competent, role model, mentor, and leader.

#### Gender and carer/competent identities

We found that participants constructed surgeons’ carer and competent identities and that these both intersected with gender. The following male patient described a consultation with a female surgeon, and in so doing, constructs the surgeon’s carer and competent identities alongside her values and attributes:

*She explained the procedure with diagrams… and explained to me exactly what would happen within the body. She was good at what she did. She was very straightforward and plain in what she was telling me… it was obvious to me what was going to happen, and she was quite comforting in that respect. But she was very capable. (ID 51, Ireland-based Male Patient, Aged 60–69).*

In this quotation, the patient uses the words “comforting” (carer identity) and “capable” (competent identity) to describe the female surgeon’s attitudes and professional capabilities. Interestingly, this quote contradicts the often-cited (but false) dualism about patients preferring a technically competent surgeon rather than one with good bedside manners (87). We see this patient constructing his female surgeon as being caring *and* competent. Female surgeons’ competence was not just commented on by patients, but was also mentioned by colleagues:

*In terms of technical skills and outcomes, the consultant [female surgeon] I worked with in my last job had as good, if not better, outcomes than anyone else in the department. And lower rates of infection than anyone else. She was … her operations took the same amount of time as anyone. I do not know where she did her fellowships or anything like that … if I could ever operate at that standard, I’d be very, very happy. (ID 71, Ireland-based Male Surgical Trainee, Aged 18–29).*

In this narrative, the male surgical trainee describes his admiration for a female surgeon employing repetition and emotive language (“*very, very happy*”). He describes this female surgeon as competent, safe, and efficient. What is interesting about these quotes, is that they construct intersecting identities of female + competent surgeon, contrasting with (mostly) female surgeons’ narratives above that question female surgeons’ competence (especially the competence of female surgeons of color, female surgeons who are mothers, and young female surgeons). While our findings support previous research reporting on competent professional identities in the context of patient preferences or coaching (45, 77), neither of these studies examined competence in relation to gender intersections among surgeons themselves.

#### Gender and mentee/mentor identities

Our female surgeon participants reported that their surgical mentors were typically male (largely because more senior male surgeons were available). This has been reported in the literature, with the need for men to be allies, sponsors and mentors for female surgeons (88). However, several flagged the limitations of male mentors for female mentees:

*… as much as I’ve had great mentors that were men… I do not think they understand the things that we, as women… have to choose between sometimes. (ID 21, Scotland-based Female Surgical Trainee, Aged 30–39).*

Describing the intersections between their female surgeon and mentee identities, female surgeons expressed a preference for female mentors who had been through similar challenges as themselves (e.g., motherhood), alluding to limitations of male mentors:

*There aren’t many role models out there of men who do not work full time … I want a woman’s perspective on it because they know what it’s like to be a mother … I think that’s probably why I feel… a mentor would be better as a woman … (ID 26, Scotland-based Female Surgical Trainee, Aged 30–39).*

This female surgical trainee would prefer a female surgical mentor who has worked part-time to look after children, expressing that she would get more from that mentoring relationship because they would better understand her. We know that mentorship has been shown to increase the likelihood of students considering a surgical career and choice of surgical specialty (89–91). We also know that lack of adequate mentoring can negatively impact women’s career progression in surgery (92). Therefore, issues relating to mentoring will continue to be challenging for female surgical trainees until surgery becomes more gender-balanced (93–97).

#### Gender and role model identities

Participants also bemoaned the lack of female role models (because of the lower numbers of female surgeons) and how this lack of gender-concordant role-modeling exacerbated “struggle” within their surgical careers:

*Actually, over the years, it’s something that frequently the trainees come to me about. They say they struggle with not having female role models in Surgery. (ID 56, Ireland-based Resigned Female Surgeon, Aged 40–49).*

This resigned female surgeon continues by suggesting that females can struggle with senior female surgical role models when they are present/available. She suggests that female surgeons who have got to the top of their career have done so through sacrificing family life and battling challenges (“tougher than the guys”), serving to normalize this suboptimal culture of surgical training:

*And there’s a feeling that the women who have made it… to the top in surgery have made these sacrifices and are tougher than the guys, and there’s almost a normalisation within the culture of that kind of sacrifice of family life if you like. (ID 56, Ireland-based Resigned Female Surgeon, Aged 40–49).*

Conversely, some male surgeons reported that female surgeons sometimes preferred a male role model:

*But then I’ve heard that in other places, females coming through surgery tend to stay away from female role models… because they find that they are more harsh on them than their male counterparts… I found that to be a very interesting one that even though there were female role models there, they did not want them; they wanted their male counterparts because they felt they’d be more fairly treated. (ID 64, Ireland-based Male Surgical Trainee, Aged 18–29).*

Although this narrative is based on a male surgical trainee’s perception, he suggests that female surgical trainees prefer to avoid having female role models because he constructs their identities as “harsh,” alluding to female-to-female violence and potentially the so-called “Queen Bee” phenomenon (98, 99). In the Queen Bee phenomenon, senior women are thought to be harsher on junior women as the seniors want to be the only woman in their immediate working environment (98). This is, however, contrary to our findings above—that female surgeons want female role models.

#### Gender and leader identities

Finally, participants constructed gender + leader identities for females in surgery. The following quotation from a female surgical trainee suggests that male surgeons/doctors mostly occupied management or leadership positions in the hospitals:

*I’ve had a few consultants that were women, but they were never heads of department or anything like that … hospital medical directors have all been men… (ID 21, Scotland-based Female Surgical Trainee, Aged 30–39).*

Although she comments that she has seen a “few” female consultant surgeons, she suggests that leadership/management roles were male-dominated. Another older female surgical trainee questions whether she would want the “extra work” entailed with management positions and suggests that other female surgeons are typically reluctant to take up leadership positions:

*… even if I was a consultant, would I want to do management*? *I probably would not… I think you have to want to do it, and it’s such a lot of extra work… But if you want something to change, I understand you have to do it… all that I’ve seen are mostly men… Although we do have female surgeons, they do not really want to do it… But I think if I [experience] something that wasn’t fair, then I would want to do it … it’s just a lot of extra work. (ID 23, Scotland-based Female Surgical Trainee, Aged 40–49).*

However, in her quotation, she hints that she might be driven to take up leadership roles if she wanted to change the status quo (e.g., tackling unfairness). Aligned with this, other participants commented that when female surgeons did take up leadership roles, they made great leaders. The following male consultant surgeon recounts his admiration for a female surgeon colleague who took up a clinical director role:

*She’s been recently appointed as the clinical director. And she’s meticulous… she’s got a very clear vision. She’s a very good communicator. She has just the right level of personal authority and collaborative spirit, so she would be an example of a fabulous leader. I do not think it’s because she’s female. I think it’s because she’s who she is. (ID 24, Scotland-based Male Consultant Surgeon, Aged 50–59).*

He identifies a raft of positive leadership characteristics exhibited by this female surgeon (e.g., meticulous, clear vision, good communicator, personal authority, collaborative). However, he explicitly disavows her gender having anything to do with these leadership capabilities (“I do not think it’s because she’s female”); instead, he credits her “fabulous” leadership to her personality (“it’s because she’s who she is,” referring to her as “she” twice in this statement).

Despite having a leadership position, several female surgeons/doctors narrated the challenges experienced by female leaders of being respected, as described in this following quotation by a female consultant anesthetist participant (talking about a female surgical leader colleague):

*I have had conversations with a colleague… around the same age as me, who is a female surgeon and was clinical lead… in a hospital where I worked. Yeah, but her experience was that the men did not listen or take her seriously at the committee level… (ID 29, Scotland-based Female Consultant Anaesthetist, Aged 50–59).*

In this quotation, the participant highlights multiple intersecting personal (female gender + mature age), and professional identities (surgeon + leader + clinically competent + incompetent leader). This highlights the multiple challenges experienced by senior female surgeons who are also leaders/managers, and might also partly account for female surgeons’ reluctance to take up leadership roles (4, 26, 77).

## Study conclusion

### Summary of key findings

Our study explored the theme of intersecting surgical identities, specifically focusing on personal (gender, ethnicity, parenthood, and age) and professional identities (carer/competent, mentee/mentor, role model, leader). Note that these constructions were articulated by surgeons themselves (mostly female), but also male and female colleagues and patients. Despite the focus of our research program on female surgeons, because we interviewed male colleagues of female surgeons, we also ended up collecting rich data on the constructions of male surgeons’ multiple identities (e.g., male + father, male + age + competence), demonstrating that female *and* male surgeons negotiated tensions between gender and parenthood identities. What our intersectionality approach highlights is that we cannot assume that all females (nor males for that matter) will have the same identities impacting their surgical lives including their education, training, and success. Indeed, this intersectionality approach in feminist theory highlights the differences between women according to other identities such as ethnicity, motherhood, and age, illustrating that a diverse array of intersecting personal and professional identities will inevitably and uniquely shape female surgeons’ professional journeys. The underlying principle applied to intersectionality is that structures of inequality are mutually constitutive, such that sexism, racism, and other “isms” co-create and reinforce each other (100). Therefore, we hope that the quotes presented above illustrate surgeons’ constructed identities in relation to their hierarchical positioning and power (or lack thereof) within the surgical environment. What our findings (talk about identities and identity talk) particularly underscore is the challenges experienced by female surgeons of color, female surgeons who are mothers, younger female surgeons, and surgical leaders, illustrating the diversity of women’s experiences extending beyond that of gender alone and adversely affecting surgical lives. Ultimately, these findings lend empirical support for intersectionality theory as a framework for understanding multiple social identities within the context of surgery (14).

### Novel contribution to the literature on identities

While some studies have previously explored the intersecting personal identities of surgeons, for example, female surgeons + motherhood and female surgeon of color (27, 42, 44–46), none have used an intersectionality lens, nor have they looked at the wide range of intersecting personal and professional identities, for example, age, competent, role model, leader, etc. To our knowledge, our study is unique in that it is the first to examine intersecting personal and professional identities within the context of surgical education, using intersectionality theory as an analytical lens. As illustrated in our combined “findings and discussion” section, our findings were reasonably consistent with existing literature, for example, indicating that women of color are more affected because of their intersecting gender + ethnic identities (25). However, our study is the first to report the challenges for female surgeons of color based in Ireland and Scotland, illustrating how intersecting gender + ethnicity identities negatively influence their interactions with patients, as well as colleagues. Our findings are also consistent with previous research illustrating intersecting gender + motherhood identities (101, 102), showing the associated motherhood penalties for female surgeons (30). However, another novel angle to our research study is the (unexpected) intersecting gender + fatherhood identities, illustrating the negative impacts of surgical cultures on male surgeons’ family lives, with them expressing their needs for more equality in terms of flexible working to enable them to embrace their fatherhood identities. We also reported intersecting gender + age identities showing that youth was perceived as disadvantageous for female surgeons (too young/inexperienced) but not for young male surgeons, a novel addition to the literature which focuses on the performance of aging surgeons and patient mortality or outcomes (31, 32). Furthermore, in relation to professional identities, our study is novel in that it explored the intersecting professional identities in relation to carer/competent, mentor/mentee, role model and leader identities, through a gendered intersectionality lens (14). Through using this theoretical lens, our study offers a more fulsome understanding of the intersecting identities of surgeons from the perspective of female surgeons across all grades of training to consultancy, including resigned female surgeons, and male and female colleagues and patients of female surgeons. This study therefore extends the current literature on intersecting personal and professional identities, which has so far focused on medical education (4), other healthcare disciplines like social work (103), medical students (27), personal identities (46) or just on professional identities (41, 44, 45).

## Methodological strengths and limitations

Our study has various methodological strengths. First, our qualitative data have sufficient information power given that we collected over 36 hours of rich, in-depth biographical narrative interview data across three participant groups (66). Second, our study explored conceptualizations in two healthcare systems, noting that we found no obvious differences in our data from participants from two English-speaking and Westernized countries. This means that our study findings are likely transferable to other English-speaking and Westernized contexts. Third, we employed a team-based approach to facilitate rigorous data analysis and interpretation, which meant that we brought diversity to the analysis, leading to a more multifaceted interpretation of the data (68).

However, our study has its limitations, which must be considered when thinking about its implications. Our first challenge relates to the transferability of our findings. While our study is likely transferable to other English-speaking and Westernized contexts, our findings in relation to ethnicity are likely to reflect the demographics of Ireland (where 17.5% of the population identify as ethnic minorities) and Scotland (where 12.9% of the population identify as ethnic minorities), so may not be transferable to other countries with different ethnicity demographics (104, 105). Our second limitation relates to several inter-related diversity issues (speciality, sample and workplaces). Although there was diversity in the number of specialities represented, the sample size from each speciality was small, so we could not explore any patterns in our data by specialty (noting that some surgical specialities, such as General Surgery, are more gender-balanced than others, such as Orthopedic Surgery). In addition, the sample sizes for the different subgroups (for example, female surgeons of color, female ex-surgeons, etc.) were similarly small, again prohibiting us from exploring patterns in our data. The number of male surgeons in our study was also small as the focus of our larger study was on female surgeons. However, the findings in relation to male surgeons’ identities were unexpected and warrant further research given younger male surgeons’ changing needs to embrace their fatherhood identities. Returning to ethnicity, all patients and non-surgical colleagues in our study were white, and thus, we advocate for future research exploring female surgeons of color, including their colleagues and patients of color. We also encourage further research on younger female surgeons, as well as identities unexplored in this paper (e.g., diverse sexualities). Moreover, given the diversity of workplaces from which our clinician participants were drawn, we were unable to make sense of their recent experiences considering their current workplaces including workplace policies on parental leave. Therefore, we recommend additional research employing institutional ethnography (observations, interviews, and documentary analysis) to further explore female surgeons’ intersecting identities contextualized to workplace structures, cultures, policies and practices. Our third challenge relates to methodological coherence. We had good internal coherence across our study philosophy, theoretical framework, methodology and methods, yet we employed thematic rather than narrative analysis. Further research, therefore, warrants more diverse types of analysis to shed light on female surgeons’ intersecting identities. Our final challenge relates to dissemination. Given our presentation of wide-ranging multiple intersecting personal and professional identities in this paper, it has been difficult to provide an in-depth interpretation of each of these intersections. Therefore, additional research should explore the more novel intersections in more analytical depth, such as male surgeons and fatherhood and resigned female surgeons’ narratives to better explore how their intersecting identities interplay with their specialty transitions.

## Study implications

Our key findings highlight that personal and professional identities are not separate but rather intersect to create opportunities and/or challenges within the surgical environment (in terms of education, training, and success). Our findings demonstrate that the unique experiences of surgeons depend on their multiple intersecting identities, meaning that a one-size-fits-all approach to any educational intervention might be suboptimal. For example, we particularly note the male surgeons’ intersecting identities (gender + fatherhood), as they could increasingly face the parenthood challenges already experienced by female surgeons. Based on the findings from our study, educational implications exist for individuals, namely surgeons, but also for colleagues and patients of female surgeons. There are also implications at the organizational level that should be considered; namely for medical schools, postgraduate training, and the workplace (see detailed recommendations in Table 2).

**Table 2:** Educational recommendations.

Surgeons	Colleagues and patients	Organization
Raise awareness in surgeons of their multiple intersecting identities and negative potential impacts on education, training, and success Provide formal training so that female and male surgeons are more aware, thoughtful, and reflexive about their multiple intersecting identities to help them cope with issues arising during their surgical training and careers	Raise awareness in colleagues and patients of the particular challenges (e.g., bias, discrimination) faced by female surgeons of color, female surgeons who are mothers, young female surgeons and female surgical leaders Provide formal training of colleagues on intersecting identities so they become more mindful (and respectful) in their dealings with female surgeons Solicit help from male colleagues as allies to continuously communicate the professional identities of female surgeons (as caring, competent, mentors, role models, leaders), rather than disavowing their female gender Consider conducting a healthcare community diversity and inclusion campaign on respecting and valuing the diversity of all staff (using female surgeons of color, female surgeons who are also mothers, young female surgeons and female surgical leaders as illustrations)	Inclusion of identities into educational and training programs, so that surgeons and colleagues develop more awareness of intersecting identities and their impacts Provide ally training for senior members of educational and healthcare organizations so that they can be better mentors and role models for female surgeons Review and potentially refine organizational policies on equality, diversity and inclusion (particularly parenthood) to expressly include an intersectionality lens

## Data availability statement

The raw data supporting the conclusions of this article cannot be made available by the authors, because they do not have ethical approval to share these data. Further inquiries can be directed to the corresponding author.

## Ethics statement

The studies involving humans were approved by the East of Scotland Research Ethics Committee (16/ES/0082), Galway University Hospital (C.A. 1,697), the Adelaide and Meath Hospital, Dublin [2017–02 CA (16)], St. James Hospital, Dublin (Expedited approval), Sligo University Hospital (Expedited approval), University Hospital Limerick (064/17), and Beaumont Hospital (17/30). The Study was conducted in accordance with the local legislation and institutional requirements. The participants provided their written informed consent to participate in this study.

## Author contributions

GO, SS, SC, and CER designed the study. GO obtained ethical approval, recruited the participants, collected the data, and wrote the manuscript. GO conducted this research as part of her Ph.D. at the Centre for Medical Education, University of Dundee, with SS, SC, and CER as her supervisors. All authors were involved in data analysis, editing, and commenting on various iterations of the manuscript.

## Footnotes

^1^For the context of our study, we have used the terminology “women of color” to denote Black and minority ethnic (BAME) women. The terminology varies across different countries and so we use terminology most accepted in the context of the study, i.e., Scotland and Ireland.

^2^The term Consultant Surgeon is employed in Ireland and the UK to mean a fully trained surgeon; equivalent to Attending Surgeon in the USA.

^3^The six female participants who had resigned from surgery made switches to other specialities (so stayed in medicine). Attrition from surgical training is not uncommon with reported attrition rates ranging from 2 to 30% over the course of surgical training (73). In England, there is a 2.68% average yearly attrition rate, while Ireland reports that 15% of those completing Basic Surgical Training do not progress to Higher Surgical Training (74, 75). Note that we were interested in including this group in our study/data analysis because of the insights that they may reveal about how their intersecting identities interplayed with their decision-making for leaving surgery.
