# Supplementary material for: Exploring constructions of female surgeons’ intersecting identities and their impacts: a qualitative interview study with clinicians and patients in Ireland and Scotland
Source: Front Med (Lausanne). 2024 Jul 22;11:1379579. doi: 10.3389/fmed.2024.1379579 (PMC11301647; doi:10.3389/fmed.2024.1379579)
Supplement: Supplementary file 1 [file Data_Sheet_1.docx]

**SUPPLEMENTARY MATERIAL**

Appendix A – Key Interview Questions for Female Surgeons

**Key Interview Questions for Female Surgeons**

1. *Please tell me the story of your life as a surgeon beginning with your medical training from medical school to postgraduate training and where relevant to surgeon consultants. Please include all events and experiences that have been important to you personally. I will not interrupt; I will just take some notes for afterwards.*

Prompts: What key experiences influenced your decision to pursue surgery? Please describe your own most memorable stories of these key experiences along your surgical path.  These experiences may be positive or negative.  Explore any challenges participants may have had as female surgeons and their perceived solutions to these challenges. Has there ever been a time that you thought about whether surgery is really for you? If so, tell me about that experience.

1. Please describe your experiences in the surgical field from the point of view of management and leadership; academic work; and teamwork and collaboration.
2. Has there ever been a time when your gender as a surgeon was commented on?

Prompts: Can you remember a time when your success was influenced by gender? Have your patients ever queried your gender?  Has there been a time as a surgeon that you felt that your other identities (i.e., age, ethnicity, race, nationality etc.) influenced your career?

1. What is your understanding of gender? What is your attitude to gender?

Prompts: What does being female mean to you? What are feminine traits? What does being male mean to you? What are masculine traits? What influences do you think gender has in medicine if any? Is medicine a gendered career?  If so, why?

1. Which do you think are male and female-dominated specialities in medicine? Why? Subsequent dialogue and prompt questions will focus on an in-depth exploration of participants’ responses. The series of prompts around participants’ narratives will include questions like: Tell me what happened? Who was involved?  Where did it happen?  When did it happen? What did you do and why? How did you feel?  What was the impact of that on your decision to become a surgeon?  Did you feel that gender influenced the situation?
